# Relationship between Differential Hepatic microRNA Expression and Decreased Hepatic Cytochrome P450 3A Activity in Cirrhosis

**DOI:** 10.1371/journal.pone.0074471

**Published:** 2013-09-13

**Authors:** Raj Vuppalanchi, Tiebing Liang, Chirayu Pankaj Goswami, Rohit Nalamasu, Lang Li, David Jones, Rongrong Wei, Wanqing Liu, Vishal Sarasani, Sarath Chandra Janga, Naga Chalasani

**Affiliations:** 1 Department of Medicine, Indiana University School of Medicine, Indianapolis, Indiana, United States of America; 2 Center for Computational Biology and Bioinformatics, Indiana University School of Medicine, Indianapolis, Indiana, United States of America; 3 Department of Medicinal Chemistry & Molecular Pharmacology, College of Pharmacy, Purdue University, West Lafayette, Indiana, United States of America; Saint Louis University, United States of America

## Abstract

**Background and Aim:**

Liver cirrhosis is associated with decreased hepatic cytochrome P4503A (CYP3A) activity but the pathogenesis of this phenomenon is not well elucidated. In this study, we examined if certain microRNAs (miRNA) are associated with decreased hepatic CYP3A activity in cirrhosis.

**Methods:**

Hepatic CYP3A activity and miRNA microarray expression profiles were measured in cirrhotic (n=28) and normal (n=12) liver tissue. Hepatic CYP3A activity was measured via midazolam hydroxylation in human liver microsomes. Additionally, hepatic CYP3A4 protein concentration and the expression of CYP3A4 mRNA were measured. Analyses were conducted to identify miRNAs which were differentially expressed between two groups but also were significantly associated with lower hepatic CYP3A activity.

**Results:**

Hepatic CYP3A activity in cirrhotic livers was 1.7-fold lower than in the normal livers (0.28 ± 0.06 vs. 0.47 ± 0.07mL* min^-1^*mg protein^-1^ (mean ± SEM), P=0.02). Six microRNAs (miR-155, miR-454, miR-582-5p, let-7f-1*, miR-181d, and miR-500) had >1.2-fold increase in cirrhotic livers and also had significant negative correlation with hepatic CYP3A activity (range of r = -0.44 to -0.52, P <0.05). Notably, miR-155, a known regulator of liver inflammation, had the highest fold increase in cirrhotic livers (2.2-fold, P=4.16E-08) and significantly correlated with hepatic CYP3A activity (r=-0.50, P=0.017). The relative expression (2^-ΔΔCt^ mean ± SEM) of hepatic CYP3A4 mRNA was significantly higher in cirrhotic livers (21.76 ± 2.65 vs. 5.91 ± 1.29, P=2.04E-07) but their levels did not significantly correlate with hepatic CYP3A activity (r=-0.43, P=0.08).

**Conclusion:**

The strong association between certain miRNAs, notably miR-155, and lower hepatic CYP3A activity suggest that altered miRNA expression may regulate hepatic CYP3A activity.

## Introduction

Cirrhosis is associated with altered drug disposition and often requires dosage adjustments in order to prevent adverse effects resulting from excessive drug/metabolite accumulation [1-4]. Although altered drug distribution and impaired renal elimination could contribute to overall drug disposition, altered activity of drug metabolizing enzymes is the predominant factor determining drug disposition [4-6]. Cytochrome P450 (CYP) 3A is the most abundant hepatic drug metabolizing enzyme and accounts for the clearance of more than 50% of those drugs that undergo biotransformation in the liver including calcium channel blockers, protease inhibitor anti-retrovirals, most statins and macrolide antibiotics [7]. Although there is considerable inter-individual variability (~ 50 fold) in the CYP3A activity in healthy humans [8], it is variably and non-uniformly reduced in cirrhosis [9-12]. Prior studies evaluating the CYP activity in cirrhosis have also shown that the loss of CYP activity is selective and dependent on the etiology (cholestatic vs. noncholestatic) and severity of liver disease [13]. George et al. showed CYP3A activity (using testosterone β-hydroxylase activity) and protein reduction only in livers of patients from cirrhosis due to non-cholestatic liver disorders [13]. Frye et al. proposed a “sequential progressive model of hepatic dysfunction” to explain their data demonstrating selective impairment of CYPs with severity of liver disease [5]. Unfortunately, CYP3A activity was not evaluated in their study [5]. Our group has previously reported a 2-fold reduction in CYP3A activity in patients with cirrhosis [14].

In healthy humans, the genetic variability in CYP3A activity could to a certain degree be explained through cis-acting elements e.g. genetic polymorphisms [8] and/or trans-acting factors such as pregnane X receptor (PXR, or nuclear receptor subfamily 1, group I, member 2 [NR1I2]) and constitutive androstane receptor (CAR, or nuclear receptor subfamily 1, group I, member 3 [NR1I3]) [15-19]. Wolbold et al. have also shown a positive correlation between PXR and CYP3A4 mRNA and protein expression in a collection of human liver biopsy tissue samples, suggesting that PXR strongly regulates the expression of CYP3A4 [20]. Some studies have suggested pre-translational regulation of CYP3A mRNA expression and activity through modulation of PXR [13,21]. Others have suggested epigenetic modification for CYP3A4 gene promoters [22]. More recently, Klein et al., have reported that peroxisome proliferator–activated receptor-α (*PPAR* α or nuclear receptor subfamily 1, group C, member 1 [NR1C1]) is also involved in the regulation of CYP3A protein in liver [23]. However, in pathological situations like cirrhosis, inflammatory signaling pathways may interfere with constitutive expression, leading to significant downregulation [24]. The exact mechanisms behind these translational or posttranscriptional regulations of hepatic CYP3A in cirrhosis are currently not well understood.

Recently discovered, microRNAs (miRNAs / miRs) are small (18-25 nucleotides), non-coding RNAs that bind to the 3’-untranslated region (3’-UTR) of mRNA and negatively regulate gene expression. These miRNAs regulate gene expression by one of the two following mechanisms - by blocking protein translation or by cleaving the mRNA and thus could at least in part contribute to post-transcriptional regulation of gene. The physiological and biological significance of miRNAs in liver function is well illustrated through down regulation of hepatocyte nuclear factor 4α (HNF4α), a key transcription factor regulation of endogenous, xenobiotic metabolizing enzymes and transporters [25-28]. Similarly, in-silico [29] and experimental analysis have also suggested that miRNAs are important regulators for CYP3A as well as PXR [21,30-34]. For example, miR27-b and mmu-miR298 decreased CYP3A4 mRNA and protein expression level in human embryonic kidney cells in transient transfection experiment [32]. Furthermore, down-regulation of vitamin D receptor (VDR) by the two miRNA led them to conclude that CYP3A4 gene expression could be regulated by miRNAs at both the transcriptional and post-transcriptional level [32]. Takagi et al. investigated the interindividual variability of CYP3A4 mRNA reported that miR-148a post-transcriptionally regulated human PXR, resulting in the modulation of the inducible and/or constitutive levels of CYP3A4 in human liver [21]. On the other hand, earlier studies profiling miRNAs in liver diseases have identified a number of miRNAs significantly up- or down-regulated in human liver diseases including cirrhosis (for review: Gastroenterology. 2012 Jun; 142(7):1431-43.), suggesting that miRNAs are critical regulators for liver function in liver disease. It is thus plausible to expect significant hepatic miRNA changes in cirrhosis that could potentially mediate the altered CYP activity, via transcriptional or translational regulations. We hypothesized in this study that the decreased CYP3A activity in cirrhosis is associated with significantly upregulated miRNAs that regulate CYP3A4 gene expression by targeting nuclear receptors (PXR, CAR or PPARα) mRNA or directly targeting CYP3A4 mRNA. The purpose of the current study is to determine the differentially expressed miRNAs in cirrhosis that are significantly associated with decreased CYP3A activity and examine the relationship with the nuclear receptor gene expression.

## Materials and Methods

### Human liver samples

This study was reviewed and approved by the Institutional Review Board (IUPUI # 0504-58 and EX0904-11) at Indiana University School of Medicine, Indianapolis, IN, USA. The institutional review board waived the need for consent. This study is based on 40 human liver tissue samples collected from individuals with cirrhosis and those with normal liver tissue (Table 1). Liver tissue was collected from individuals with established cirrhosis (n=28) at the time of their liver transplantation procedure in the operating room. Demographic data, etiology of cirrhosis and other relevant information such as medication, alcohol use, and smoking history were captured at the time of enrollment. Normal liver tissue (n=12) was obtained from patients undergoing liver resection for metastatic/benign liver lesions with no underlying chronic liver disease. Histopathological evaluation with hematoxylin and eosin staining was used to assess tumor adjacent normal liver tissue in order to exclude occult chronic hepatitis and cirrhosis. Liver tissue samples were snap frozen using liquid nitrogen and stored at -80°C until use. All samples were collected and handled equally except for duration of storage.

**Table 1:** Select demographics, gene expression and CYP3A activity in study cohort.

	**Cirrhosis** (n=28)	**Normal** (n=12)	
**Demographics**			
Age, years (mean ± SD)	57.4 ± 13.7	50.5 ± 18.8	
Females (%)	50	42	
Non-Hispanic white (%)	96	100	
BMI kg/m^2^ (mean ± SD)	27.6 ± 5.7	Not available	
Diabetes (%)	46	Not available	
History of smoking (%)	32	Not available	
Etiology of cirrhosis (%)			
- NASH (n=17)	61	N/A	
- Cholestatic (n=11)	39	N/A	
Concomitant HCC (n=5)	18	N/A	
**Relative gene expression** (2^-ΔΔCt^ mean ± SEM)			**Pvalue**
CYP3A4 mRNA	21.76 ± 2.65	5.91 ± 1.29	2.04E-07
PXR mRNA	6.15 ± 0.45	4.45 ± 0.87	0.04
CAR mRNA	6.26 ± 0.43	3.55 ± 0.71	0.0008
PPARα mRNA	0.63 ± 0.06	0.06 ± 0.01	1.90029E-12
**CYP3A activity** (mL*min^-1^*mg protein^-1^) (mean ± SEM)	0.28 ± 0.06	0.47 ± 0.07	0.02

N/A: not applicable

### CYP3A activity in cirrhosis and normal liver tissue

Liver homogenates were prepared and suspended in buffer and stored in a -80°C freezer until use. The protein concentration of the homogenates was assayed using Lowry method [35]. Hepatic microsomes were prepared as published previously [35] and stored at −80°C until used. The CYP3A activity was quantified in human liver microsomes using midazolam 1’-hydroxylation as probe reaction. In brief, midazolam (0.25-75 µmol/L) was incubated with human liver microsomes (0.25-800 µg total protein), 100 mmol/L phosphate buffer pH = 7.4, and 2 mmol/L reduced nicotinamide adenine dinucleotide phosphate for 2 minutes. The reaction was stopped with an equal volume of acetonitrile. 1’-OH midazolam was quantified from the supernatant with HPLC-UV (λ = 254) using alprazolam as the internal standard. The maximum velocity of metabolite formation (V_max_, pmols*mg protein^-1^*min^-1^), and the Michaelis constant (K_m_, µM) were estimated using a single enzyme model with WinNonlin Standard Edition v2.0 (Pharsight, Palo Alto CA). In vitro intrinsic clearance (CL_int_) of CYP3A was given as V_max_ / K_m._

### miRNA microarray

Global microRNA expression analysis (485 miRNA probes passed statistical filtering) was performed by Thermo Scientific Dharmacon RNAi Discovery and Therapeutic Services (Lafayette, Colorado, USA). The Thermo Scientific Dharmacon microRNA expression profiling platform utilizes a proprietary, two-color high-density 8-plex slide comprised of probes to capture all human, mouse and rat mature microRNAs in the Sanger database (http://microrna.sanger.ac.uk). The microRNA expression data has been uploaded to Gene Expression Omnibus archive and is set for data release as of August 15^th^ 2013 (Accession number GSE49012).

### Confirmation of candidate microRNA and quantification of mRNA expression using qRT-PCR

Total RNA including miRNA was isolated from the liver tissue using mirVana™ miRNA isolation Kit (Life Technologies, Grand Island, NY). To eliminate residual genomic DNA contamination, the RNA samples were incubated with DNase I (RNase-Free DNase Set, QIAGEN) on columns according to the manufacturer’s instructions. The RNA quantity and quality were measured using both Nano drop and Agilent bioanalyzer to ensure the good quality of RNA. The amplification primers for PXR, CAR and PPARα were designed using *Vector NTI*. With the ABI 7300 qPCR system, TaqMan chemistry was used for microRNA quantification and the SybrGreen was applied to quantify mRNA levels. Following reverse transcription of the RNA (Life Technologies), an aliquot of each reverse transcription reaction was amplified in triplicate in an ABI 7300 qPCR system. This system generates quantitative data based on the PCR at early cycles when amplification is in the linear range and PCR fidelity is the highest. GAPDH and 18s rRNA were used as internal controls for mRNA quantification and U6 was used as internal control for miRNA quantification. We quantified CYP3A4, PXR, and CAR mRNA and miRNA levels using comparative method (ΔΔCT method) and PPARα mRNA levels using relative standard curve method (Life Science htpp://www.appliedbiosystems.com) [36,37].

### Western Blotting of CYP3A4

Randomly selected cirrhotic (n=5) and normal liver samples (n=5) were used to measure the hepatic CYP3A4 protein expression. Briefly, 10mg of liver tissue was homogenized using the Mini-Beadbeater-16 (Biospec Products, OK, USA). The homogenates were centrifuged and the supernatant was collected. Total protein concentration of each supernatant sample was measured using Qubit® 2.0 Fluorometer (Life Technologies, CA, USA) and normalized to same concentration prior to western blotting. For western blotting, 500ng total protein of each sample was separated using 12% SDS-PAGE gel electrophoresis, transferred to nitrocellulose membranes, and then blotted with anti-CYP3A4 (XenoTech, KS, USA) and anti-GAPDH (Abcam, MA, USA) as an internal control. The blotted proteins were detected and visualized using the ECL Western Blotting kit (Amersham Biosciences, NJ, USA). Quantification of the intensity of blotted band was conducted using ImageJ (http://rsbweb.nih.gov/ij/). The relative expression of CYP3A4 was determined by normalizing the CYP3A4 band intensity to that of GAPDH of the same sample.

### Statistical and bioinformatics analyses

Batch effect in the miRNA microarray samples was removed statistically. ANOVA analysis with False Discovery Rate (FDR) and multiple test correction were performed to identify differentially expressed miRNAs between cirrhotic and control liver tissue samples. Comparisons of mRNA expression were made between groups by using Student’s t-test. Pearson’s correlation test was utilized to assess the correlation between miRNA expression and CYP3A activity and between mRNA expression of PXR, CAR or PPARα and CYP3A4 mRNA. Nonparametric Mann-Whitney test was used to compare the difference in the relative CYP3A4 protein expression between cirrhotic and normal livers. Results from correlation analysis and ANOVA analysis were merged to identify a subset of differentially expressed miRNAs which are significant upregulated in the cirrhotic group (P<0.05) and yet had significant negative correlation with CYP3A activity (P<0.05). A P-value <0.05 was considered statistically significant.

To predict the potential binding sites of the miRNAs identified to control the CYP3A4 gene in this study, we first obtained the transcripts encoded by this gene’s locus. Current annotations from the Ensembl [38] suggest that two major transcripts ENST00000336411 and ENST00000354593 are responsible for the protein expression of CYP3A4. So we obtained the 3’ UTR sequences of these transcripts using Ensembl biomart. The 3’ UTR sequence of ENST00000336411 comprised of 464 base pair while that of ENST00000354593 was 1172 base pair in length. Fasta sequences of 14 microRNAs found to correlate with CYP3A activity were obtained from miRbase [39]. Genomic targets for microRNAs were predicted by using microRNA Target detection software miRanda [40]. Miranda is a machine learning method for ranking microRNA target sites by a down-regulation score by integrating gene expression and sequence level features. The algorithm trains a regression model on sequence and contextual features extracted from high confidence miRanda-predicted target sites and hence can predict non-canonical and non-conserved sites. Target sites and their locations were identified for the miRNAs of interest using the max score threshold set to 110. A total of 55 sites were identified on the 3’ UTRs of these two transcripts. Target sites predicted by miRNA were formatted into a General Feature Format (GFF) file using the binding co-ordinates with respect to the UTR regions, to visualize the location of the binding sites using GFF2PS tool [41].

## Results

### Hepatic CYP3A activity and protein expression

**Figure 1:**
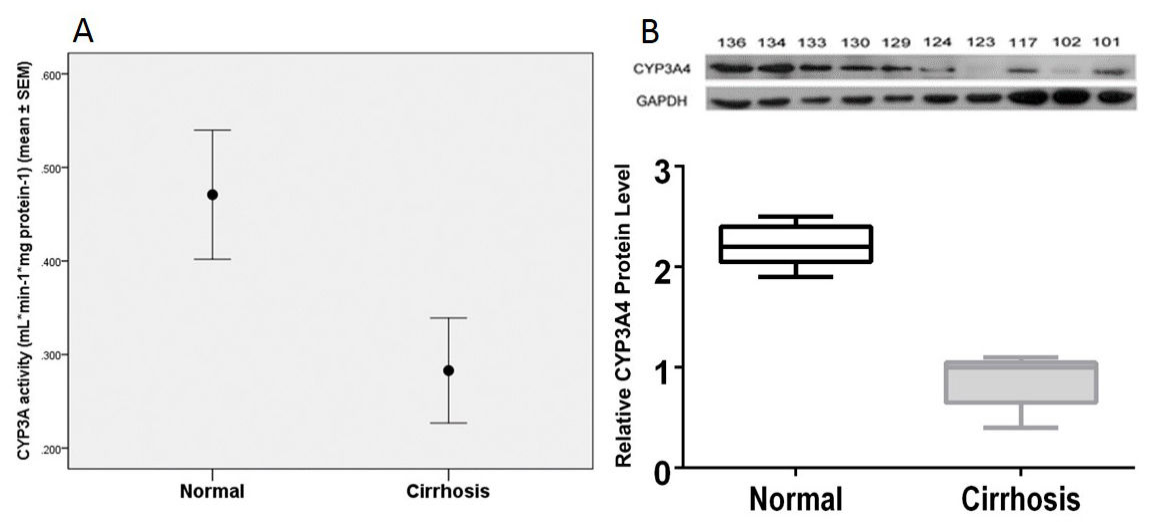
Cytochrome P450 3A activity and protein expression in cirrhosis and normal liver tissue samples. (**A**) Decreased CYP3A activity as determined by the intrinsic clearance based on formation of 1’-OH midazolam formation in cirrhosis and normal liver tissue samples. (**B**) CYP3A4 protein expression in randomly selected cirrhotic (n=5) and normal liver samples (n=5) detected by western blotting (upper panel). Quantified CYP3A4 expression relative to GAPDH was shown as well (bottom panel). Experiments were repeated twice and the representative sample was shown.

Hepatic CYP3A activity in cirrhotic liver samples was significantly lower compared to normal liver tissue by 1.7-fold (0.28 ± 0.06 vs. 0.47 ± 0.07mL* min^-1^*mg protein^-1^ (mean ± SEM), P=0.02) (Figure **1A**). Similarly, CYP3A protein level in cirrhotic liver samples was significantly lower than in control liver samples by western blotting (P=0.01) and a quantitative image analysis showed a 2.5-fold lower level of CYP3A protein in cirrhotic livers (Figure **1B**).

### Differential hepatic miRNA expression and their relationship to hepatic CYP3A activity

**Figure 2:**
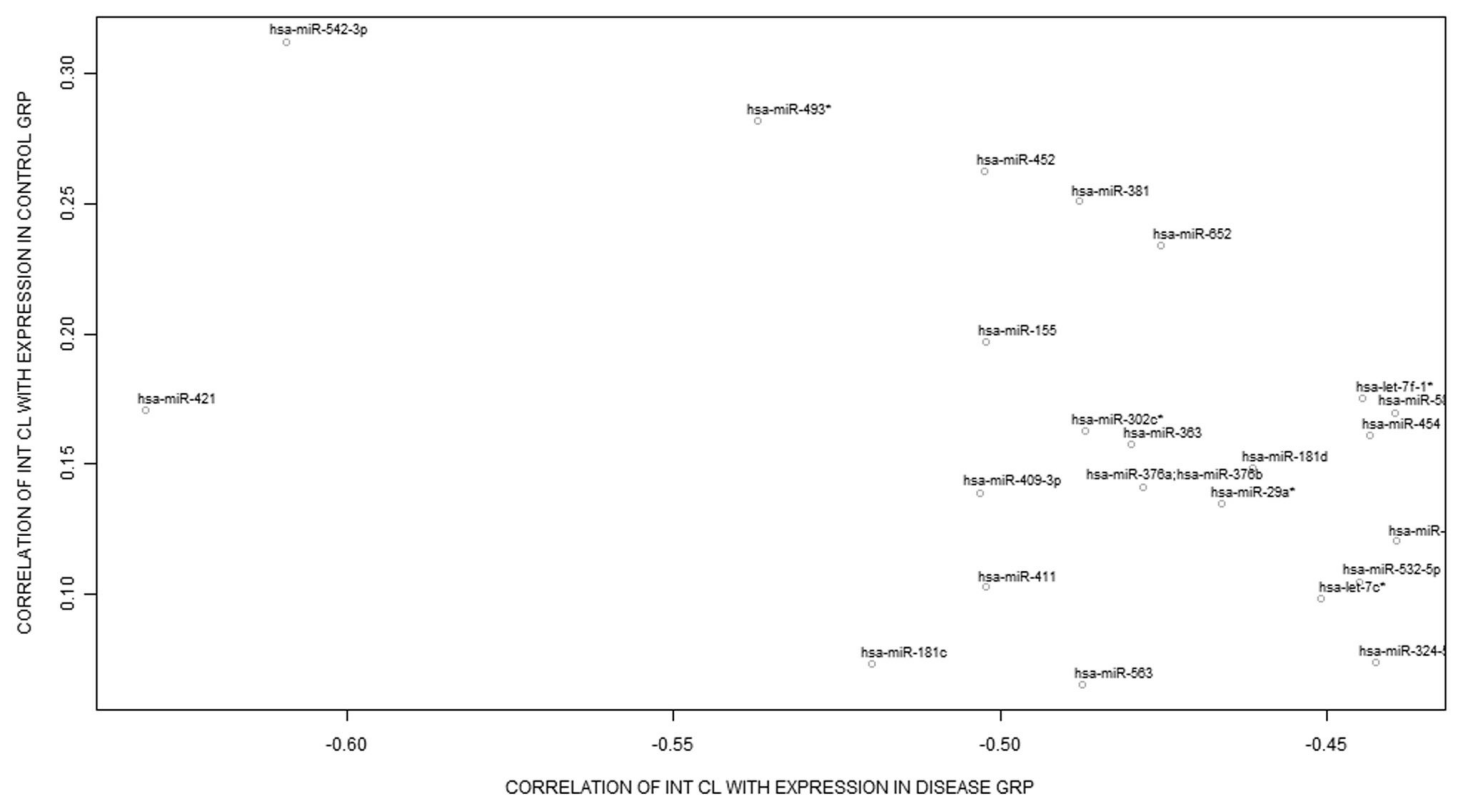
Correlation between correlation of expression of selected miRNA with CYP3A activity (X-axis) in cirrhosis group and the correlation of the expression of the same miRNA with CYP3A activity in the normal liver group (Y-axis). Each point in the figure represents a single miRNA.

A total of 183 miRNAs were differentially expressed in cirrhotic liver tissue compared to normal liver tissue with P<0.05 and a fold-change ranging from -2.47 to 2.27. Several miRNAs had significant correlation with hepatic CYP3A activity and also had significant differential expression between cirrhotic and control liver tissues (Figure **S1**). To generate a list of miRNAs that potentially regulated hepatic CYP3A activity, we merged the results of the ANOVA and correlation analyses which identified a subset of miRNAs with significantly higher expression in cirrhotic livers and also had significant negative correlation with hepatic CYP3A activity (Figure **2**). This merged analysis revealed 14 miRNAs with higher expression in cirrhotic livers but had negative correlation with hepatic CYP3A activity (Table **2**). Among the miRNAs, miR-155, miR-454, miR-582-5p, let-7f-1*, miR-181d, and miR-500 had > 1.2-fold increase in cirrhotic liver samples (Table **2**). Of particular interest is miR-155 which had the highest fold increase in cirrhotic livers (2.2-fold, P=4.16E-08) and negatively correlated with CYP3A activity (r = -0.50, P=0.017). There was a significant negative correlation between mean expression of all 14 miRNAs combined and hepatic CYP3A activity (r = -0.53, P=0.012).

**Table 2:** miRNAs that are upregulated in cirrhotic livers and had significant negative correlation with hepatic CYP3A activity.

**miRNA**	**Correlation (r value**)** of miR expression with CYP3A activity**	**P (for differential miRNA expression**)	**Fold-Change (cirrhosis/control**)^¶^	
			**Microarray**	**qRT-PCR**
hsa-miR-155	-0.50	4.16E-08	2.2	3.6
hsa-miR-454	-0.44	2.29E-06	1.5	2.6
hsa-miR-582-5p	-0.44	7.47E-06	1.4	1.4
hsa-let-7f-1*	-0.44	9.73E-05	1.4	1.6
hsa-miR-181d	-0.46	0.000212	1.3	1.8
hsa-miR-500	-0.44	0.000412	1.3	1.3
hsa-miR-181c	-0.52	0.005571	1.2	NA
hsa-miR-411	-0.50	0.008032	1.2	NA
hsa-miR-532-5p	-0.44	0.011412	1.2	NA
hsa-miR-363	-0.48	0.016422	1.2	NA
hsa-miR-381	-0.49	0.017241	1.2	NA
hsa-miR-302c*	-0.49	0.030258	1.2	NA
hsa-miR-652	-0.47	0.036603	1.2	NA
hsa-miR-452	-0.50	0.040303	1.1	NA

NA: No data available as qRT-PCR was only performed on top 6 microRNA.

The fold change of hepatic miRNA in cirrhosis is in comparison to healthy liver control tissue samples.

¶ The fold change of hepatic miRNA in cirrhosis is in comparison to healthy liver control tissue samples.

### Relationship between CYP3A4 and selected nuclear receptor gene expression

The CYP3A4, PXR, CAR and PPARα mRNA were detected in all liver samples and they exhibited significant variability in their expression both in cirrhotic and normal liver tissue (Table **3**). Their relative expression was significantly higher in cirrhotic livers compared to normal liver samples (Table **3**). To investigate the association between selected nuclear receptors (PXR, CAR and PPARα) and *CYP3A4* gene expression, their correlation was examined in cirrhotic and control groups separately. CYP3A4 mRNA tightly correlated with PXR mRNA (r=0.91, P= 2.50E-08), CAR mRNA (r=0.93, P= 5.42E-09) and PPARα mRNA (r=0.92, P= 9.56E-09) in the control group. Although not as stronger, this relationship was still apparent in cirrhotic livers where CYP3A4 mRNA correlated with PXR (r=0.46, P=0.01), CAR (r=0.51, P=0.006) and PPARα (r=0.54, P=0.003) mRNA expression.

**Table 3:** CYP3A4, PXR, CAR and PPARα mRNA expression in cirrhotic and normal liver samples.

	**Intra-group variability in mRNA expression**		**Relative mRNA expression**		
	**Normal (n=12**)	**Cirrhosis (n=28**)	**Normal (n=12**)	**Cirrhosis (n=28**)	**P-value**
**CYP3A4 mRNA**	449-fold	209-fold	5.91 ± 1.29	21.76 ± 2.65	2.04E-07
**PXR mRNA**	490-fold	14-fold	4.45 ± 0.87	6.15 ± 0.45	0.04
**CAR mRNA**	172-fold	24-fold	3.55 ± 0.71	6.26 ± 0.43	0.0008
**PPARα mRNA**	3637-fold	225-fold	0.06 ± 0.01	0.63 ± 0.06	1.90029E-12

### Relationship between selected nuclear receptors mRNA, CYP3A4 mRNA and CYP3A activity

The negative correlation between CYP3A4 mRNA and hepatic CYP3A activity in cirrhotic liver samples failed to reach statistical significance (r = -0.43, P=0.08). In cirrhotic liver samples, there was generally a negative correlation between CYP3A activity and CAR, PXR and PPARα mRNA expression, but the association was significant only for PXR mRNA (r= -0.54, P=0.02). Interestingly, in the normal liver tissue there was no significant association between hepatic CYP3A activity and CYP3A4, CAR, PXR or PPARα mRNA expression.

### Relationship between miRNAs and mRNA expression of selected nuclear receptors mRNA and CYP3A4 mRNA

In order to examine the potential role of the selected 14 miRNA in the regulation of hepatic CYP3A activity through post-transcriptional regulation, a correlation analysis was performed in normal and cirrhosis groups separately between miRNA and mRNA expression of CYP3A4 and selected nuclear receptors. In the normal liver tissue, the majority of miRNA correlated with mRNA expression of CYP3A4 (57%) and nuclear receptors (up to 93% for PPARα). In contrast, this degree of correlation was not seen in cirrhosis (Table **4**). Only two miRNA (has-let 7f 1* and has-miR 500) correlated with CYP3A4 mRNA and only one miRNA (has-miR 181d) correlated with PXR mRNA.

**Table 4:** Correlation analysis between miRNA and mRNA expression of CYP3A4 and select nuclear receptors (PXR, CAR and PPARα) in cirrhosis and normal liver tissue.

**Cirrhosis**								
	***CYP 3A4*** ******		***PXR***		***CAR***		***PPARα***	
**Normal**	**R**	**P**	**R**	**P**	**R**	**P**	**R**	**P**
hsa_miR_302c_*	0.39	0.084364	-0.20	0.387729	-0.10	0.663752	-0.36	0.109445
**hsa_miR_582_5p**	0.36	0.107842	-0.15	0.512426	0.02	0.937971	-0.23	0.322844
hsa_let_7f_1_*	0.44	***0.044578***	-0.23	0.316745	-0.10	0.657649	-0.32	0.151573
**hsa_miR_411**	0.41	0.067978	-0.27	0.232043	-0.17	0.473656	-0.11	0.63548
**hsa_miR_381**	0.32	0.156986	-0.19	0.397427	-0.06	0.797608	0.03	0.901204
**hsa_miR_454**	0.40	0.068732	-0.41	0.067509	-0.25	0.267514	-0.26	0.248468
**hsa_miR_181c**	0.26	0.260728	-0.27	0.244541	-0.17	0.471892	-0.08	0.7299
**hsa_miR_181d**	0.40	0.069606	-0.44	***0.048326***	-0.34	0.137086	-0.13	0.566524
**hsa_miR_155**	0.37	0.101412	-0.31	0.173238	-0.20	0.38348	-0.16	0.480846
**hsa_miR_532_5p**	0.42	0.057871	0.00	0.988572	0.19	0.41248	-0.22	0.340639
**hsa_miR_500**	0.47	***0.031564***	-0.13	0.571989	-0.02	0.927534	-0.16	0.483486
**hsa_miR_652**	0.39	0.080473	-0.23	0.31141	-0.10	0.653354	-0.13	0.574028
**hsa_miR_363**	0.34	0.128097	-0.16	0.475747	-0.06	0.800896	-0.04	0.873588
**hsa_miR_452**	0.32	0.15923	-0.04	0.861106	0.14	0.539199	-0.20	0.382359
hsa_miR_302c_*	-0.61	**0.036424**	-0.65	**0.021117**	-0.73	**0.007031**	0.82	**0.001124**
**hsa_miR_582_5p**	-0.59	**0.044251**	-0.64	**0.024298**	-0.68	**0.014715**	0.70	**0.011959**
hsa_let_7f_1_*	-0.60	**0.038018**	-0.66	**0.019887**	-0.71	**0.00951**	0.76	**0.004071**
**hsa_miR_411**	-0.57	0.053899	-0.62	**0.032519**	-0.70	**0.011777**	0.80	**0.001961**
**hsa_miR_381**	-0.55	0.065207	-0.59	**0.042576**	-0.57	**0.051956**	0.48	**0.110354**
**hsa_miR_454**	-0.48	0.114549	-0.57	0.053069	-0.63	**0.027302**	0.72	**0.007791**
**hsa_miR_181c**	-0.59	**0.043219**	-0.61	**0.034777**	-0.70	**0.011955**	0.83	**0.000837**
**hsa_miR_181d**	-0.59	**0.04374**	-0.61	**0.03656**	-0.68	**0.014522**	0.79	**0.002116**
**hsa_miR_155**	-0.50	**0.099561**	-0.53	0.075015	-0.60	**0.038651**	0.67	**0.016024**
**hsa_miR_532_5p**	-0.57	0.050579	-0.61	**0.03519**	-0.67	**0.018061**	0.76	**0.0041**
**hsa_miR_500**	-0.55	0.066793	-0.59	**0.043274**	-0.65	**0.021733**	0.73	**0.007229**
**hsa_miR_652**	-0.65	**0.023005**	-0.71	**0.009294**	-0.78	**0.002854**	0.82	**0.001057**
**hsa_miR_363**	-0.52	0.084987	-0.56	0.057266	-0.64	**0.02412**	0.74	**0.00571**
**hsa_miR_452**	-0.77	**0.003249**	-0.80	**0.001671**	-0.85	**0.000485**	0.83	**0.000736**

### In silico verification of CYP3A binding by miRNAs

**Figure 3:**
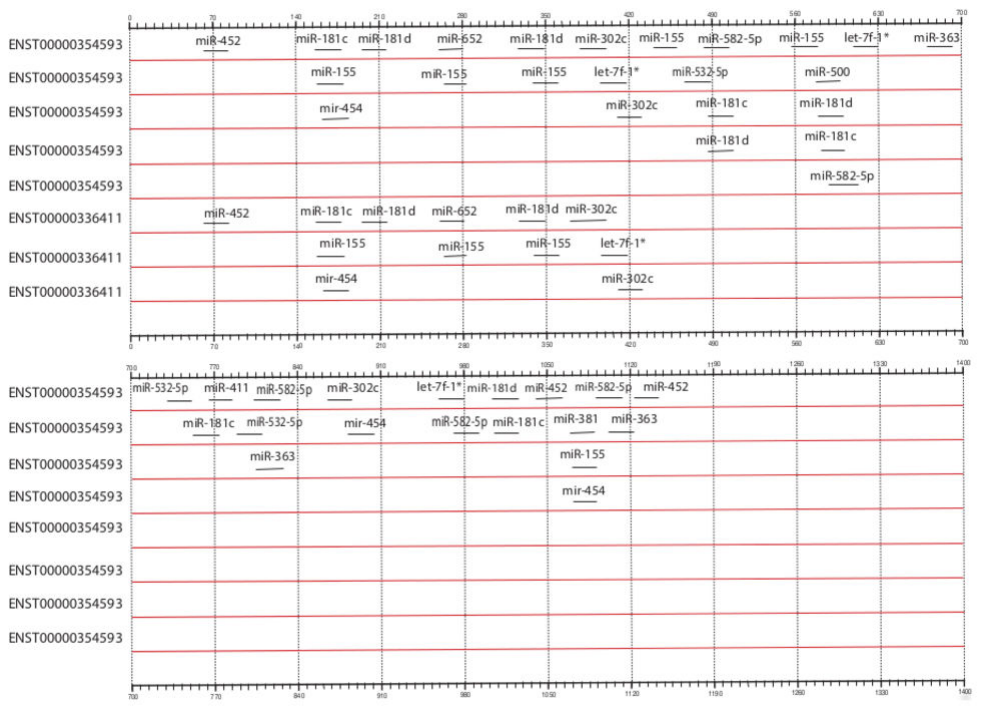
Block diagram showing the distribution of miRNA binding sites in the 3’ Untranslated regions (UTR) of the CYP3A4 transcripts. UTR regions of the both the transcripts ENST00000354593 (length 1172 bp) and ENST00000336411 (474 bp) are aligned to the left and shown from 5’ to 3’ orientation. When multiple miRNAs were found to overlap in their binding locations on the respective transcripts, different tracks are used to improve the layout.

In this study, to identify the binding locations of the 14 miRNAs found to be associated with CYP3A4 gene, we used miRanda algorithm [40]. A total of 55 binding sites were identified in the 3’ UTRs of the CYP3A4 transcripts (see Table S1 and Figure 3). Figure 3 shows the distribution of these binding sites across the UTRs of the two transcripts. While 14 miRNAs were detected to have a binding site in the UTR of the longer transcript ENST00000354593, a total of 8 miRNA binding sites were detected in the untranslated regions of the second transcript.

## Discussion

It has been long recognized that hepatic CYP3A activity is decreased in cirrhosis and this reduced hepatic CYP3A activity is a very clinical relevant issue because nearly 50% of all prescribed medicines are CYP3A substrates. But it is not well understood why cirrhosis is associated with lower hepatic CYP3A activity. Intuitively, one might consider decreased hepatic synthesis of CYP3A protein as the most likely explanation for this phenomenon, but there has not been a consistent relationship between hepatic CYP3A mRNA expression and hepatic CYP3A protein concentration or CYP3A activity. We examined if hepatic miRNAs influence hepatic CYP3A activity in cirrhosis and in fact we found evidence to suggest that certain miRNAs may play a role in the pathogenesis of decreased hepatic CYP3A activity in cirrhosis. The main observations are our study are: (a) it confirms that cirrhosis is associated with lower hepatic CYP3A activity as well as protein concentration; (b) some miRNAs are associated with lowered hepatic CYP3A activity in cirrhosis; and (c) the expression of CYP3A4 mRNA as well as a number of nuclear receptors (PXR, CAR and PPARα) known to regulate CYP3A expression is higher in cirrhosis. Based on these observations we speculate that cirrhosis is associated with increased expression of certain miRNAs which interfere with translation of CYP3A4 mRNA and synthesis of CYP3A protein. There may be a resulting compensatory increase in the expression of nuclear receptors and CYP3A4 mRNA. The increase in the expression of nuclear receptors and CYP3A4 mRNA is suggestive of post-transcriptional regulation of hepatic CYP3A activity by miRNA in cirrhosis.

We observed 14 miRNAs which had significantly higher expression in cirrhotic livers but also had significant negative correlation with hepatic CYP3A activity (Table 2). Computational methods have played an important role in the prediction of miRNAs from the very beginning. Traditionally, some major features such as the hairpin-shaped stem loop structure, high minimal folding free-energy, gene expression relationships between miRNA-mRNA pairs and high evolutionary conservation of the seed regions have been used in the computational identification of miRNAs. A previous study from our institution had predicted eleven of these fourteen (79%) miRNAs (hsa-miR-454, has-miR-582-5p, has-miR-181d, has-miR-500, has-miR-181c, has-miR-411, has-miR-363, has-miR-381, has-miR-302c*, has-miR-652 and has-miR-452), to target CYP3A4/5/7 3’-UTR using in silico approach [29]. In this study, to identify the binding locations of the 14 miRNAs found to be associated with CYP3A4 gene, we used miRanda algorithm [40]. A total of 55 binding sites were identified in the 3’ UTR of the CYP3A4 transcripts (Figure 3). While 14 miRNAs were detected to have a binding site in the UTR of the longer transcript ENST00000354593, a total of 8 miRNA binding sites were detected in the untranslated regions of the second transcript. We found several combinations of miRNAs including miRNA-155, miRNA-181 and miRNA-652 as well as miRNA-363, miRNA-532 and miRNA-582 which clustered or overlapped in their binding locations on these UTRs, suggesting possible competition for binding to control gene expression in a combinatorial fashion.

Importantly, several of the differentially expressed miRNAs have previously been suggested to have a role in the pathogenesis of liver fibrosis. Of significant interest are miRNAs with pro-fibrotic effect (miR-32, miR-155 and miR-15b*) exhibiting an increased expression, and miRNAs with an anti-fibrotic effect (miR-18a, miR-18*, miR-19a*, miR-19b-1*, miR-200a* and miR-335*) showing lower expression in cirrhotic livers (data not shown) [42-44]. Among these differentially regulated microRNA, miR-155 appears to be the most prominent regulator as it was significantly associated with lower hepatic CYP3A activity and > 2-fold higher expression in the cirrhotic livers. Previous studies have shown miR-155, a regulator of inflammation, to be increased in different types of chronic liver diseases such as alcoholic liver disease, drug induced liver injury and hepatocellular cancer [45-48]. Our study is unable to discern why miR-155 and miR-32 (and other 12 miRNAs) are overexpressed in cirrhosis, but it is possible that underlying etiological factors (e.g., hepatitis C, alcohol, insulin resistance), hepatic inflammation or liver fibrosis may play a role. Our findings suggest that inhibiting these miRNAs by antisense oligonucleotides might be an approach to correct hepatic CYP3 dysregulation in cirrhosis, although admittedly the clinical relevance of such a strategy is debatable.

Certain aspects of our study need further discussion. Our study used microarrays to investigate the miRNAs and we admit that next generation high-throughput, high-resolution sequencing technology may discover new miRs and bring added value, but the costs were still prohibitive when this study was planned. Currently, microarray (microchip) profiling and quantitative real-time reverse transcription PCR (qRT-PCR) are the two most common methods. However, the results from microarray and qRT-PCR do not always concur. In order to address this concern, the top 5 candidate miRNAs from the microarray profiling were further confirmed by qRT-PCR to validate the accuracy of our microarray findings (Table 2). Second, CYP3A5 mRNA was not measured in the current study. However, it is unlikely that the differential expression of CYP3A5 mRNA would affect our conclusions since its contribution to total liver CYP3A activity is minor [49]. Moreover, subjects enrolled in our study are all but one non-Hispanic Caucasian and are less likely to express CYP3A5 *1 allele which leads to substantially expression of CYP3A5 protein and greater contribution to the total CYP3A activity. Our study could not confirm the previously reported relationship between miR-27b, mmu-miR298 and miR-148a and CYP3A activity. We speculate that difference in the study design i.e. human liver tissue expression vs. cell line transfection experiments may account for the disparity. In addition to miRNA regulation, post-transcriptional/translation regulation may also be due to the altered protein synthesis, modification and/or trafficking which we have not investigated in this study.

## Conclusions

CYP3A activity is significantly lower in cirrhosis and was associated with differential expression of hepatic miRNA. The significant association between decreased CYP3A activity and up-regulation of select hepatic miRNA in cirrhosis is strongly suggestive of their regulatory role. Our study points towards miR-155 and miR-32 as two potential miRNA which may a play in the pathogenesis of decreased hepatic CYP3A activity in cirrhosis.

## Supporting Information

Figure S1 Correlation between correlation of expression of miRNA with CYP3A activity i.e. intrinsic clearance (X-axis) in disease (cirrhosis) group and the correlation of the expression of the same miRNA with CYP3A activity in the control (normal) liver group (Y-axis).Each point in the figure represents a single miRNA. The size of the point represents that significance of the correlation in the cirrhosis group (there was no significant correlation of these miRNA with the CYP3A activity in the healthy livers). Left upper quadrant of the figure shows all the miRNAs which have positive correlation with CYP3A in healthy group (Y-axis), and negative correlation in the disease group (X-axis). The miRNAs in blue color are upregulated in the cirrhosis group, and those in red color are down regulated in expression. Circles represent miRNAs whose expression is statistically significantly correlated with CYP3A activity while triangles represent miRNA with no significant correlation. Thus, miRNA of interest for the study are those in the orange color, biggest in size and represented in the left upper quadrant.
(TIF)

Table S1 List of miRNA target sites for CYP3A4 gene.The binding locations of the miRNAs are shown with respect to the UTR co-ordinates of the respective transcripts.
(DOCX)
