# Supplementary figures and images for: Relationship between Differential Hepatic microRNA Expression and Decreased Hepatic Cytochrome P450 3A Activity in Cirrhosis
Source: PLoS One. 2013 Sep 13;8(9):e74471. doi: 10.1371/journal.pone.0074471 (PMC3772944; doi:10.1371/journal.pone.0074471)

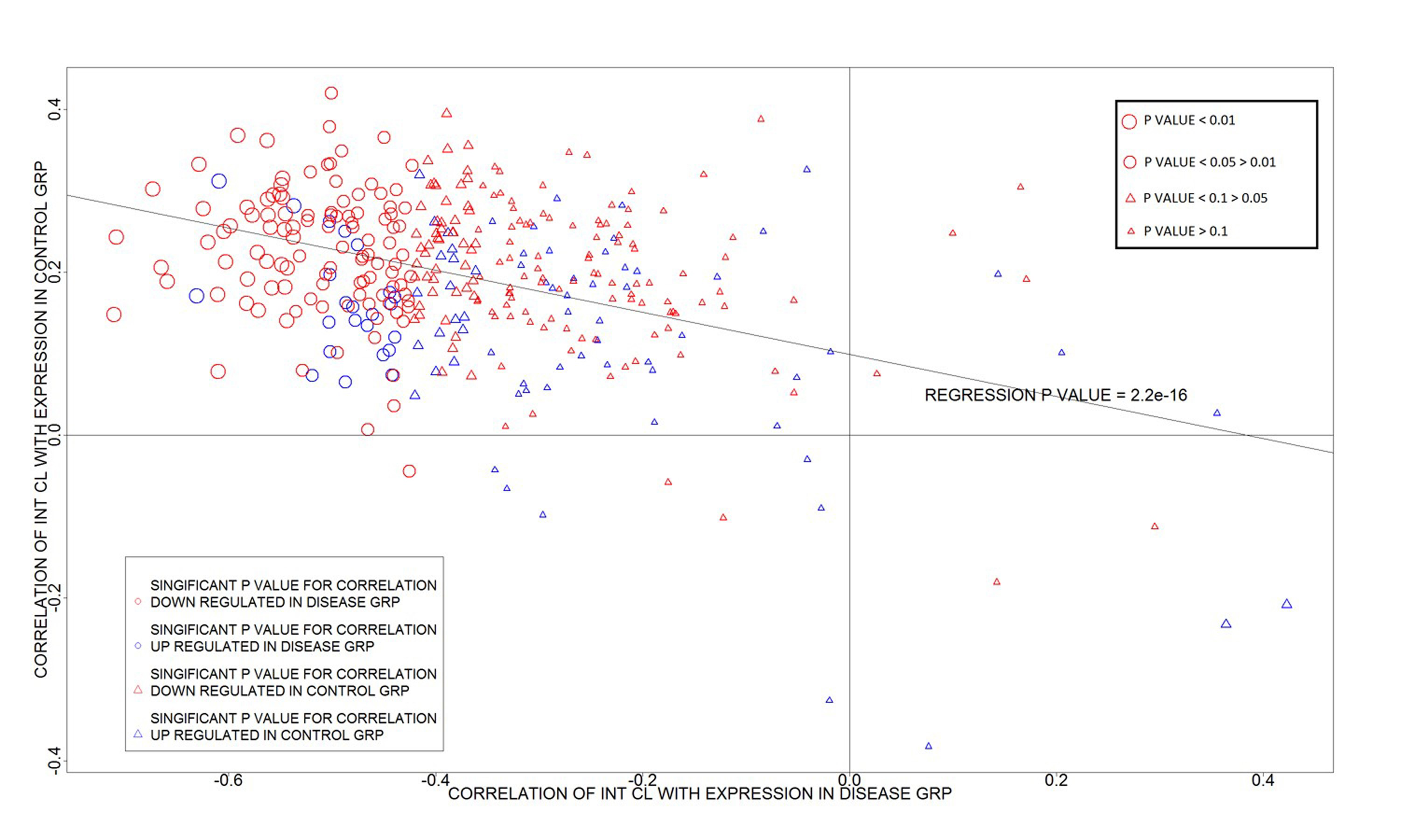

Supplement: Figure S1 — Correlation between correlation of expression of miRNA with CYP3A activity i.e. intrinsic clearance (X-axis) in disease (cirrhosis) group and the correlation of the expression of the same miRNA with CYP3A activity in the control (normal) liver group (Y-axis).Each point in the figure represents a single miRNA. The size of the point represents that significance of the correlation in the cirrhosis group (there was no significant correlation of these miRNA with the CYP3A activity in the healthy livers). Left upper quadrant of the figure shows all the miRNAs which have positive correlation with CYP3A in healthy group (Y-axis), and negative correlation in the disease group (X-axis). The miRNAs in blue color are upregulated in the cirrhosis group, and those in red color are down regulated in expression. Circles represent miRNAs whose expression is statistically significantly correlated with CYP3A activity while triangles represent miRNA with no significant correlation. Thus, miRNA of interest for the study are those in the orange color, biggest in size and represented in the left upper quadrant. (TIF) [file pone.0074471.s001.tif]
