# Supplementary material for: Relationship between Differential Hepatic microRNA Expression and Decreased Hepatic Cytochrome P450 3A Activity in Cirrhosis
Source: PLoS One. 2013 Sep 13;8(9):e74471. doi: 10.1371/journal.pone.0074471 (PMC3772944; doi:10.1371/journal.pone.0074471)
Supplement: Table S1 — List of miRNA target sites for CYP3A4 gene.The binding locations of the miRNAs are shown with respect to the UTR co-ordinates of the respective transcripts. (DOCX) [file pone.0074471.s002.docx]

**Table S1. List of miRNA target sites for CYP3A4 gene. The binding locations of the miRNAs are shown with respect to the UTR co-ordinates of the respective transcripts.**

| **Gene** | **Transcript** | **MicroRNA** | **Max Score** | **Max _energy** | **Start** | **Stop** |
| --- | --- | --- | --- | --- | --- | --- |
| CYP3A4 | ENST00000354593 | hsa-let-7f-1* | 117 | -5.99 | 959 | 980 |
| CYP3A4 | ENST00000354593 | hsa-let-7f-1* | 114 | -2.55 | 396 | 418 |
| CYP3A4 | ENST00000354593 | hsa-let-7f-1* | 113 | -5.6 | 609 | 630 |
| CYP3A4 | ENST00000354593 | hsa-miR-155 | 133 | -10.65 | 441 | 461 |
| CYP3A4 | ENST00000354593 | hsa-miR-155 | 121 | -3.87 | 339 | 361 |
| CYP3A4 | ENST00000354593 | hsa-miR-155 | 117 | -15.4 | 557 | 579 |
| CYP3A4 | ENST00000354593 | hsa-miR-155 | 116 | -10.63 | 157 | 180 |
| CYP3A4 | ENST00000354593 | hsa-miR-155 | 115 | -16.12 | 1071 | 1092 |
| CYP3A4 | ENST00000354593 | hsa-miR-155 | 112 | -11.97 | 264 | 283 |
| CYP3A4 | ENST00000354593 | hsa-miR-181c | 129 | -10.37 | 486 | 508 |
| CYP3A4 | ENST00000354593 | hsa-miR-181c | 124 | -10.97 | 582 | 601 |
| CYP3A4 | ENST00000354593 | hsa-miR-181c | 119 | -19.85 | 156 | 178 |
| CYP3A4 | ENST00000354593 | hsa-miR-181c | 115 | -6.33 | 1005 | 1026 |
| CYP3A4 | ENST00000354593 | hsa-miR-181c | 111 | -17.74 | 752 | 774 |
| CYP3A4 | ENST00000354593 | hsa-miR-181d | 139 | -10.15 | 486 | 508 |
| CYP3A4 | ENST00000354593 | hsa-miR-181d | 125 | -10.2 | 579 | 601 |
| CYP3A4 | ENST00000354593 | hsa-miR-181d | 111 | -9.67 | 195 | 216 |
| CYP3A4 | ENST00000354593 | hsa-miR-181d | 110 | -13.7 | 327 | 349 |
| CYP3A4 | ENST00000354593 | hsa-miR-181d | 110 | -9.73 | 1004 | 1026 |
| CYP3A4 | ENST00000354593 | hsa-miR-302c* | 146 | -10.62 | 410 | 431 |
| CYP3A4 | ENST00000354593 | hsa-miR-302c* | 131 | -10.31 | 865 | 886 |
| CYP3A4 | ENST00000354593 | hsa-miR-302c* | 114 | -9.35 | 379 | 401 |
| CYP3A4 | ENST00000354593 | hsa-miR-363 | 120 | -9.09 | 1102 | 1123 |
| CYP3A4 | ENST00000354593 | hsa-miR-363 | 114 | -17.6 | 805 | 828 |
| CYP3A4 | ENST00000354593 | hsa-miR-363 | 112 | -14.25 | 671 | 693 |
| CYP3A4 | ENST00000354593 | hsa-miR-381 | 114 | -6.77 | 1069 | 1090 |
| CYP3A4 | ENST00000354593 | hsa-miR-411 | 120 | -10.23 | 765 | 785 |
| CYP3A4 | ENST00000354593 | hsa-miR-452 | 140 | -9.92 | 1123 | 1144 |
| CYP3A4 | ENST00000354593 | hsa-miR-452 | 117 | -12.46 | 62 | 83 |
| CYP3A4 | ENST00000354593 | hsa-miR-452 | 116 | -12.52 | 1041 | 1063 |
| CYP3A4 | ENST00000354593 | hsa-miR-454 | 141 | -15.23 | 882 | 905 |
| CYP3A4 | ENST00000354593 | hsa-miR-454 | 128 | -11.99 | 1072 | 1091 |
| CYP3A4 | ENST00000354593 | hsa-miR-454 | 114 | -14.25 | 162 | 184 |
| CYP3A4 | ENST00000354593 | hsa-miR-500 | 120 | -9.41 | 577 | 598 |
| CYP3A4 | ENST00000354593 | hsa-miR-532-5p | 120 | -15.19 | 789 | 810 |
| CYP3A4 | ENST00000354593 | hsa-miR-532-5p | 113 | -20.14 | 467 | 489 |
| CYP3A4 | ENST00000354593 | hsa-miR-532-5p | 112 | -16.01 | 730 | 751 |
| CYP3A4 | ENST00000354593 | hsa-miR-582-5p | 125 | -7.79 | 1091 | 1113 |
| CYP3A4 | ENST00000354593 | hsa-miR-582-5p | 115 | -7.64 | 971 | 993 |
| CYP3A4 | ENST00000354593 | hsa-miR-582-5p | 112 | -12.41 | 588 | 613 |
| CYP3A4 | ENST00000354593 | hsa-miR-582-5p | 112 | -16.93 | 803 | 826 |
| CYP3A4 | ENST00000354593 | hsa-miR-582-5p | 110 | -5.58 | 483 | 505 |
| CYP3A4 | ENST00000354593 | hsa-miR-652 | 130 | -17.58 | 260 | 281 |
| CYP3A4 | ENST00000336411 | hsa-let-7f-1-3p | 114 | -2.55 | 396 | 418 |
| CYP3A4 | ENST00000336411 | hsa-miR-155-5p | 121 | -3.87 | 339 | 361 |
| CYP3A4 | ENST00000336411 | hsa-miR-155-5p | 116 | -10.63 | 157 | 180 |
| CYP3A4 | ENST00000336411 | hsa-miR-155-5p | 112 | -11.97 | 264 | 283 |
| CYP3A4 | ENST00000336411 | hsa-miR-181c-5p | 119 | -19.85 | 156 | 178 |
| CYP3A4 | ENST00000336411 | hsa-miR-181d | 111 | -9.67 | 195 | 216 |
| CYP3A4 | ENST00000336411 | hsa-miR-181d | 110 | -13.7 | 327 | 349 |
| CYP3A4 | ENST00000336411 | hsa-miR-302c-5p | 146 | -10.62 | 410 | 431 |
| CYP3A4 | ENST00000336411 | hsa-miR-302c-5p | 114 | -9.35 | 370 | 401 |
| CYP3A4 | ENST00000336411 | hsa-miR-452-5p | 117 | -12.46 | 62 | 83 |
| CYP3A4 | ENST00000336411 | hsa-miR-454-3p | 114 | -14.25 | 162 | 184 |
| CYP3A4 | ENST00000336411 | hsa-miR-652-3p | 130 | -17.58 | 260 | 281 |
